# Urban and Rural Differences in Logistics When Attending In-Person Appointments

**DOI:** 10.1093/geroni/igaf122.912

**Published:** 2025-12-31

**Authors:** Elizabeth Chamberlin, Steven Shirk, Victoria Ngo, Elizabeth Marfeo, Lauren Moo

**Affiliations:** VA Bedford Health Care System, Bedford, Massachusetts, United States; VA Bedford Health Care System, Bedford, Massachusetts, United States; VA Bedford Health Care System, Bedford, Massachusetts, United States; Tufts University, Medford, Massachusetts, United States; VA Bedford Healthcare System, Bedford, Massachusetts, United States

## Abstract

The lack of long-term formal care for older adults and the wish to age in place has increased the use of informal caregivers. These caregivers take on many responsibilities, including assisting their care recipients with in-person healthcare visits. A national survey of informal caregivers of military Veterans (N = 511), included the VAL to assess logistical differences between urban (n = 253) and rural (n = 258) caregivers when assisting in in-person health care appointments. There was no significant difference between urban and rural Veterans’ level of functioning. For the six domains of VAL (1) scheduling, 2) preparing for the visit, 3) visit-related time and travel, 4) work, therapeutic, academic, and recreational impact, 5) routine disruption, and 6) overall feelings/impression), preparing (2), time (3), and impact (4) were significantly different for urban and rural caregivers. Gathering veterans’ information (e.g., complete forms, medication lists) (p = .02) and helping the Veteran prepare for the appointment beyond their usual routine (p =.04) took rural caregivers significantly more time than urban caregivers. Time to get to the appointment (p=.03), as well as the total round-trip time for all trips (e.g., dropping off at childcare, picking up Veteran) (p=.003) and total distance (p = <.0001) was significantly greater for rural caregivers than their urban counterparts. Rural caregivers also missed more work (p=.003) than urban caregivers. These findings suggest that rural caregivers need additional assistance to deter burnout. Increasing programs and services supporting informal caregivers in assisting care recipients is necessary.

